# Race‐Based Humor and Peer Group Dynamics in Adolescence: Bystander Intervention and Social Exclusion

**DOI:** 10.1111/cdev.12600

**Published:** 2016-09-29

**Authors:** Kelly Lynn Mulvey, Sally B. Palmer, Dominic Abrams

**Affiliations:** ^1^ University of South Carolina; ^2^ University of LondonUCL Institute of Education; ^3^ University of Kent

## Abstract

Adolescents’ evaluations of discriminatory race‐based humor and their expectations about peer responses to discrimination were investigated in 8th‐ (*M*_age_ *= *13.80) and 10th‐grade (*M*_age_ *= *16.11) primarily European‐American participants (*N *= 256). Older adolescents judged race‐based humor as more acceptable than did younger adolescents and were less likely to expect peer intervention. Participants who rejected discrimination were more likely to reference welfare/rights and prejudice and to anticipate that peers would intervene. Showing awareness of group processes, adolescents who rejected race‐based humor believed that peers who intervened would be more likely to be excluded. They also disapproved of exclusion more than did participants who supported race‐based humor. Results expose the complexity of situations involving subtle discrimination. Implications for bullying interventions are discussed.

Research on prejudice, bias, and discrimination indicates the early emergence of negative intergroup attitudes and relations (Aboud, 2005; Nesdale, 2008), which are often deeply entrenched by adulthood (Dovidio, Hewstone, Glick, & Esses, 2010). The effects of discrimination and prejudice on children's and adolescents’ healthy development are numerous. For instance, adolescents who report experiencing bias‐based discrimination, such as discrimination based on their group membership, are at high risk for academic, mental health, and substance abuse problems (Russell, Sinclair, Poteat, & Koenig, 2012). Furthermore, although research increasingly points to the positive role that cross‐group friendships can have for youth (Bagci, Rutland, Kumashiro, Smith, & Blumberg, 2014; Graham, Munniksma, & Juvonen, 2014), discriminatory experiences can have quite negative effects on cross‐ethnic relations (Yip & Douglass, 2011). What is not known, however, is how adolescents evaluate race‐based discrimination from within their peer group. Furthermore, little is known about whether their awareness of, or desire to participate in, maintaining the group norm may affect other group members’ responses to instances of race‐based discrimination, such as potential interventions that challenge such discrimination. The current study focuses on these phenomena during adolescence because this is a developmental period marked by an awareness of race‐based discrimination (Wong, Eccles, & Sameroff, 2003) and greater capacity to understand the biased and prejudicial treatment of others based on group membership (Brown & Bigler, 2005; Killen, Mulvey, & Hitti, 2013). Furthermore, during adolescence there is both a heightened awareness of and challenge posed by coming to terms with the social meaning of one's racial group membership as a social identity (Rivas‐Drake et al., 2014). Finally, during this time period, adolescents increasingly attend to issues of group functioning and prioritize group norms and loyalty to the group (Killen, Rutland, Abrams, Mulvey, & Hitti, 2013). Thus, adolescence is a critical developmental period for addressing the gap in the literature regarding evaluations of and responses to race‐based discrimination in the context of peer group dynamics.

The current study examines this gap by drawing on research from subjective group dynamics (Abrams & Rutland, 2008) and social domain theory (Turiel, 1983). Specifically, research from subjective group dynamics indicates the powerful influence that group norms can have on judgments in group contexts (Abrams & Rutland, 2008). For instance, as children approach adolescence they develop “group nous”—an awareness that peers sometimes expect and value in‐group bias because such bias is viewed as a normative expression of loyalty to the group (Abrams, 2011; Abrams, Rutland, Pelletier, & Ferrell, 2009). Importantly, regardless of their personal beliefs or preferences, as children approach adolescence they become more keenly aware of, and responsive to, the weight that peers place on sustaining in‐group norms. Thus, an important focus for this theory is perceptions of how peers will respond to individuals who behave in normative versus counternormative ways.

Social domain theory focuses particularly on the ways individuals’ reason about actions when faced with complex social choices that pit the needs or rights of different individuals against one another. Social domain theory proposes that individuals consider three distinct domains of social knowledge in making judgments: (a) the moral domain, which involves issues of rights, justice, and welfare; (b) the societal domain, which involves issues surrounding customs, traditions, conventions, and group functioning; and (c) the psychological domain, which involves personal choice and autonomy (Smetana, 2006). Research drawing on both social domain theory and subjective group dynamics has documented that children and adolescents support group members who deviate from unfair group norms, such as those condoning unequal allocation of resources (Killen, Rutland, et al., 2013), but, despite this, they remain concerned about being excluded from the group for deviating from such group norms (Hitti, Mulvey, Rutland, Abrams, & Killen, 2014). In the context of gender stereotypes, research indicates that adolescents and, even more so, children are willing to challenge group norms even though they recognize that this may lead to social exclusion from the group (Mulvey & Killen, 2015). The current study extends prior research by focusing on how adolescents evaluate group norms that condone subtle forms of discrimination, for instance those supporting race‐based humor, and how they expect their peers to react to instances of subtle discrimination.

Peer groups may hold norms that condone prejudicial or discriminatory behavior (Abrams, 2011; Rutland, Killen, & Abrams, 2010). Such group norms can encourage behaviors such as excluding others based on group membership (Killen, Mulvey, et al., 2013), bullying and victimization (Russell et al., 2012), and prejudicial treatment of others (Nesdale, 2011). Often, however, these behaviors can be quite subtle or indirect. For example, microaggressions are subtle statements or behaviors that communicate harmful messages about one's group (e.g., assumptions of inferiority or criminality based on group membership; see Nadal, 2011). Experiencing these subtle forms of discrimination is related to greater reports of anxiety, depression, and stress (Huynh, 2012). Race‐based humor, although not traditionally considered a microaggression, also communicates denigrating, discriminatory messages about racial/ethnic groups. Race‐based humor occurs frequently (Ford & Ferguson, 2004) and research indicates that racial/ethnic teasing and joking has negative impacts for adolescents in terms of stress (Edwards & Romero, 2008) and anxiety (Douglass, 2014).

Race‐based humor is particularly interesting because of the role humor plays in creating positive affect and social bonds through shared implicit meanings (Romero & Pescosolido, 2008). Thus, humor usually serves a positive function for group cohesiveness, but the content of the humor may be deeply prejudicial, promote discrimination, or be damaging to particular individuals or groups (Ford, Triplett, Woodzicka, Kochersberger, & Holden, 2014; Nowakowski & Antony, 2013). Upon hearing race‐based humor, then, the listener faces a dilemma between reinforcing group norms and cohesion and tacitly accepting an undesirable or inappropriate attitude. Thus, the current study focuses on race‐based humor because it is a common form of subtle discrimination and one to which we expected adolescents would express a variety of responses. Some adolescents may judge the discriminatory behavior as acceptable, potentially because of concerns with not disrupting group norms or because they infer that acknowledging a racial component within humor may reflect negatively on them; appearing “color‐blind” is a concern shown to be increasingly likely with age (Apfelbaum, Pauker, Ambady, Sommers, & Norton, 2008). Still others may judge race‐based humor to be unacceptable due to an awareness of the harmful nature of prejudice and discrimination (Rutland & Killen, 2015).

Importantly, although bystander intervention is not all that common (Hawkins, Pepler, & Craig, 2001) and has been shown to decline further with age (Palmer, Rutland, & Cameron, 2015; Rigby & Johnson, 2006), research indicates that when bystanders act to help bullied peers they can have a powerful influence in reducing instances of negative peer interactions, such as bullying and teasing. Specifically, research on bystander intervention suggests that schools where students report that they defend victims of bullying show reduced rates of bullying (Salmivalli, Voeten, & Poskiparta, 2011). Interestingly, bystanders intervene only 25% of the time, but when they do, the bullying tends to stop within 10 seconds (Hawkins et al., 2001). Research specifically suggests that bystanders can be especially helpful in instances of intergroup bullying or discrimination, such as ethnic name calling (Aboud & Joong, 2008). Research also indicates that children and adolescents with higher levels of intergroup contact are more likely to intervene in instances of ethnic name calling (Abbott & Cameron, 2014). Additionally, research indicates that a majority of students report hearing discriminatory teasing (weight based, in this study) and that many students, especially those who have been trained to attend to prejudicial messages, also support intervening in such instances of discrimination (Paluck, 2011). Importantly for the current study, research has shown that defending behavior not only decreases into adolescence (Palmer et al., 2015; Rigby & Johnson, 2006) but that perceiving a norm for helping can reduce this developmental effect in the intergroup context of direct verbal aggression (Palmer et al., 2015). However, little is known about how adolescents evaluate bystander intervention when doing so would explicitly conflict with a peer group norm condoning the behavior, whether evaluations of bystander interventions differ by age, and how adolescents evaluate a quite subtle form of discrimination: race‐based humor.

Intervening in an instance of race‐based humor may require both a recognition of the harm that the humor can cause as well as the willingness to challenge the peer group. Although adolescents may recognize discrimination as morally wrong, and adults have been shown to regard overt racism as crossing a moral threshold relative to other types of transgressions (Abrams, Travaglino, Randsley de Moura, & May, 2014), intervening can be difficult because members who challenge the peer group's norms risk being rejected by those peers (Abrams & Rutland, 2008). Indeed, children's awareness of being observed by fellow group members heightens their motivation to adhere to salient norms of loyalty to the in‐group (Abrams, Rutland, Cameron, & Ferrell, 2007). Research indicates that children and adolescents do understand that while challenging the peer group is important, in some instances, it can also lead to social consequences, such as exclusion from the peer group (Mulvey & Killen, 2015). Intervening may pose a paradoxical challenge for adolescents. Although they may recognize the moral objections to race‐based humor, they may also be aware of the potential social costs of objecting to it, for instance social exclusion. Therefore, the difficulty of reconciling a critical private response and a noncritical public response may result in inaction or withdrawal.

## Current Study

The current study aims to examine this tension between moral and group‐serving action in adolescents, to clarify what types of bystander responses adolescents expect from peers, and to identify their expectations of the social consequences for challenging race‐based humor (about African Americans or Latinos) in terms of social exclusion. We measured both race‐based humor targeting African American and Latino peers as research suggests that Latino and African American adolescents report similar rates of intergroup discrimination by their peers (Rosenbloom & Way, 2004) and as these groups make up the predominant ethnic out‐groups in the region of the Southeastern United States where the data were collected (U.S. Census Bureau, 2015, June 16). We chose to examine both 8th and 10th graders as peer group dynamics change during adolescence: Prior research indicates that 10th graders may be especially attuned to the importance of showing loyalty to the group (Rutland, Hitti, Mulvey, Abrams, & Killen, 2015). This research extends the prior literature by examining peer group dynamics in relation to group norms that encourage race‐based humor and by examining expectations both about peer interventions and the social consequences of such interventions. Participants were told that their group norm condoned humor, including humor about different groups of people. They heard about a group member who tells a race‐based joke and were asked to make a moral judgment: They rated the acceptability of this act. Importantly, no overt victim or authority figure was present while the joke was told, but the joke was heard by several in‐group peers. This was to ensure that participants focused on the humor itself within a group context and not only the potential victimization.

They also learned about a group member who did not agree with the race‐based humor and participants were asked to assess the likelihood of this dissenting member engaging in different possible responses. Given research that indicates low rates of actual bystander intervention as well as research documenting that individuals predict that they would be more likely to challenge stereotypic group norms than they expect that their peers would (Mulvey & Killen, 2015), we chose to focus on expectations of peer responses as opposed to expectations of individual responses. Measuring expectations about a peer's responses may be a more authentic representation of actual intervention behavior than would judgements about one's own expected responses. Participants evaluated a range of different response behaviors, drawn from the prior literature on bystander intervention (Abbott & Cameron, 2014; Palmer et al., 2015; Salmivalli et al., 2011). Some responses implicitly condoned the group norm and the race‐based humor, such as staying with the group and not speaking out. Others explicitly supported the group norm (e.g., laughing) or challenged the group norm (e.g., telling the group to stop joking or talking to the joker).

Finally, given prior research which suggests that children and adolescents may not challenge group norms out of concerns about social exclusion from their peer group (Hitti et al., 2014; Mulvey & Killen, 2015), participants were asked to consider the likelihood that this dissenting member would be excluded from the group for intervening and the acceptability of such exclusion. Both descriptive (likelihood of exclusion) and evaluative (acceptability of exclusion) measures were included as research indicates that children readily reject exclusion based on moral reasons but that they recognize that exclusion often does occur based on reasons associated with group membership (Killen & Rutland, 2011).

Based on prior research that indicates that, with age, children adhere more strongly to group norms and attend to issues of group functioning (Horn, 2003; Killen, Rutland, et al., 2013), we expected that, with age, participants would be more likely to condone race‐based humor. Research also documents differences between 8th and 10th graders in their attention to issues of group loyalty: 10th graders reference group loyalty more frequently when justifying decisions related to peer group dynamics than do children or younger adolescents (Rutland et al., 2015). Furthermore, we expected that participants who condoned the race‐based humor would use different forms of reasoning than participants who did not condone the humor. Specifically, because of their desire to avoid moral tainting of the group (Abrams et al., 2014), we expected that those who condoned the race‐based humor would be less likely to use moral reasoning (such as references to prejudice and welfare) than those who rejected the race‐based humor. Additionally, given the increasing pressure to conform to groups during adolescence (Brechwald & Prinstein, 2011) and previous research on bystander intentions (Palmer et al., 2015), we expected that with age participants would be less likely to expect peers to intervene in instances of race‐based humor and more likely to expect that their peers would exclude someone who did choose to challenge race‐based humor.

We expected that participants who thought the humor was unacceptable would perceive the distinction between their personal view and the group norm more sharply, making the group norm itself more salient (see Marques, Abrams, Paez, & Hogg, 2001). Thus, they may be more sensitive to the possibility that someone who transgressed the norm by intervening would be excluded by the group. However, they would be less likely to condone such exclusion themselves. Given that research indicates that adolescents refer to group functioning when justifying social exclusion and that they also evaluate exclusion as wrong for moral reasons (Killen & Rutland, 2011), we expected that participants would focus primarily upon group functioning when justifying likelihood of exclusion and primarily on welfare/rights when justifying acceptability of exclusion. Given prior research that suggests that children may be concerned about social exclusion as a consequence for challenging group norms (Mulvey & Killen, 2015), we also expected that likelihood of types of responses would predict likelihood of exclusion.

Given the frequent lack of gender findings in moral development research on peer group dynamics, we did not expect differences based on participant gender (Killen, Rutland, et al., 2013). It was an open question whether there would be differences in evaluations and reasoning between participants who evaluated humor targeting Latinos and those who evaluated humor targeting African Americans. On the one hand, research indicates that Latino and African American youth perceive instances of discrimination differently (Seaton, Neblett, Cole, & Prinstein, 2013). However, research also reports that Latino and African American adolescents report similar rates of intergroup discrimination by their peers (Rosenbloom & Way, 2004).

## Method

### Participants

Participants (*N = *256) included 8th‐ (*M*_age_ **=13.80, *SD *= 0.79) and 10th‐grade (*M*_age_ *= *16.11, *SD *= 0.58) students. Participants were primarily European‐American adolescents (92.5%), approximately equally divided by gender (55% female) and from two schools in the same community in the Southeastern United States serving predominantly European‐American students (over 85% European American). At the high school, 42% of students were eligible for free and reduced meals, and at the middle school, 23% of students were eligible for free and reduced meals. The data were collected during the fall of 2013 and spring of 2014. All participants received parental consent and provided assent.

### Design

The *race‐based humor task* included two versions which varied based on whether the humor targeted Latino or African American out‐group members. There were no differences based on version, thus all analyses were collapsed across version. Participants were introduced to their group of friends who shared their ethnic/racial background. They chose a name and a symbol for the group. Next, participants learned that their group holds a group norm which condones humor about different groups of people and about a group member (gender matched) who tells a race‐based joke. For example, a female participant in the Latino target version would read:

Your group enjoys telling each other jokes about lots of things, including about different groups of people. Now, imagine that the school day has not yet started and you are hanging out with your group of friends in the hallway. There are no teachers around. Brittany, who is one of the kids in your group of friends, tells a joke about Latino people. There are no Latino students around right now. Because your group enjoys telling jokes about lots of things, including about different groups of people, the group finds it funny and starts to laugh.

They were also told about a dissenting member (gender matched), who did not condone race‐based humor. A female who received the African American target version would read:

Katie, who is also in your group of friends, wants to be different from the rest of your group. Katie thinks your group shouldn't tell jokes about people who are African American.

Participants completed items that assessed their moral judgments about the race‐based humor, their expectations for how their peers would respond to the humor, and their judgments surrounding exclusion of someone who challenged the race‐based humor (see Measures, below).

### Procedure

The tasks were administered by a trained researcher in a quiet room at each school. Participants were given a warm‐up task, which involved practising using the Likert‐type scale to be used in the survey. The survey was administered by a trained researcher to larger groups (25–30 participants). Most participants completed the survey electronically, on school‐provided iPads. Paper surveys were provided when electronic access was not available. Participants recorded their answers. Any questions the participants had were answered by the researcher. The survey took about 25–30 min for each participant to complete.

### Measures

#### Dependent Measures

Measures included (1) *acceptability of race‐based humor*: How okay or not okay is X for making this joke? (Likert‐type: 1* *= *really not okay* to 6 = *really okay*); and (2) *likelihood of types of response*: How likely or not likely [do] you think it is that Y will… engage in Z response? (Likert‐type: 1 = *definitely would not* to 6 = *definitely would*). Potential responses were (a) explicitly support (“Join in with the joke”), (b) implicitly support (“Not get involved and stay with the group”), (c) explicitly challenge (an average of three statements: “Walk away,” “Tell the group they shouldn't tell jokes about X people,” and “Talk to X about it after”), and (d) implicitly challenge (an average of two statements: “Get help from a teacher, family member or other adult” and “Get help from a friend”).

Participants were told that a dissenting member chose to tell the group that it was not okay to use race‐based humor. Next, participants assessed exclusion items modified from Mulvey and Killen (2015) and Killen, Rutland, et al. (2013): (3) *likelihood of exclusion*: How likely or not likely is it that your group will tell Y…that s/he cannot sit with them at lunch? (Likert‐type: 1 = *definitely would not* to 6 = *definitely would*) and (4) *acceptability of exclusion*: How okay or not okay is it for your group to tell Y s/he cannot sit with them at lunch? (Likert‐type: 1 = *really not okay* to 6 = *really okay*). Participants also provided reasoning justifications (Why?) for *acceptability of race‐based humor* and both *exclusion* measures.

#### Coding Categories for Justifications

A coding system was established based on pilot testing, and drawing on prior research (Killen, Rutland, et al., 2013). The coding system, which was used to code all justification data, included two broad categories, based on social domain theory (Smetana, 2006): moral and societal. The subcategories for *moral* were welfare/rights (e.g., “It will hurt someone's feelings if you tell mean jokes about them” or “It's not ok to tell her she can't sit with you at lunch; That will hurt her feelings”) and prejudice (e.g., “It's racist to tell jokes like that”). The subcategories for *societal* were group functioning (e.g., “The group will work better if they all agree” or “He doesn't agree with them, so eating lunch together will just mess up the group”) and importance (e.g., “It's not a big deal” or “It doesn't really matter if you tell a joke. No one cares”). Justifications were also coded for the psychological domain, but they were used infrequently (under 3% of participants referenced psychological justifications across the different measures), and thus, were not analyzed. Justifications were coded as 1 = *full use of the category*, 0.5 = *partial use*, 0 = *no use of the category* and analyses were conducted on proportional usage of each type of reasoning. Surveys were coded by three trained research assistants and interrater reliability was high (25% of surveys, *N *=* *64), Cohen's κ = .94.

### Data Analytic Plan

Data were analyzed using analysis of variance (ANOVA) and repeated measures analyses of variance (ANOVAs) to test hypotheses for between‐group differences, using age group, and a dichotomous version of the *acceptability of race‐based humor* judgments (created using a midpoint split of 3.5 in order to compare those who judged the humor to be acceptable [*N *=* *80] to those who judged it be unacceptable [*N *=* *176]). The participants, overall, found race‐based humor unacceptable (*M *=* *2.69, *SD *= 1.44). The repeated measures factors varied depending on the hypothesis, but included type of reasoning or assessment. Follow‐up tests were conducted using univariate ANOVAs or the Bonferroni correction to control for Type I errors. Analyses for the hypothesis regarding relations between the possible responses and the *likelihood of exclusion* were conducted using multiple regression. Justifications were proportions of responses for each respective coding category (see footnote 4, Wainryb, Shaw, Laupa, & Smith, 2001), with the top justifications analyzed using repeated measures ANOVAs. As gender and version (Latino or African American target) were not significant, both were dropped from the analyses. Cell sizes were > 32, ensuring power of 0.9 or greater to detect small to medium effect sizes.

## Results

### Acceptability of Race‐Based Humor

In order to test the hypothesis that with age participants would be more likely to condone race‐based humor, a 2 (age group: 8th, 10th grade) univariate ANOVA was conducted on *acceptability of race‐based humor*. As expected, a main effect of age group was found: *F*(1, 254)* *= 20.30, *p *<* *.001, $\eta_{p}^{2}=.07$. Eighth graders (*M *= 2.34, *SD *= 1.22) were less likely to condone the race‐based humor than were 10th graders (*M *= 3.13, *SD *= 1.56).

### Acceptability of Race‐Based Humor: Reasoning

In order to test the hypothesis that participants who condoned the race‐based humor would use different forms of reasoning than participants who did not condone the humor, a 2 (age group: 8th, 10th) × 2 (acceptability of race‐based humor: okay, not okay) × 3 (reasoning: welfare/rights, prejudice, and importance) ANOVA was conducted with repeated measures on the last factor. A main effect for reasoning was found: *F*(2, 504)* *= 28.27, *p *<* *.001, $\eta_{p}^{2}=.10$. This revealed that participants used more references to welfare/rights and to the low importance of the humor than to prejudice (see Table 1). Furthermore, there was an interaction between reasoning and age group: *F*(2, 504)* *= 5.02, *p *= .008, $\eta_{p}^{2}=.02$. Participants did not differ in their use of reasoning about prejudice: (*M*_8th_ **= 0.16, *SD *= 0.33; *M*_10th_ **= 0.13, *SD *= 0.29). However, younger participants were more likely to reference welfare/rights than were older participants (*p < *.001; *M*_8th_ **= 0.49, *SD *= 0.43; *M*_10th_ **= 0.33, *SD *= 0.39) and were less likely to reference importance than were older participants (*p < *.001; *M*_8th_ **= 0.18, *SD *= 0.33; *M*_10th_ **= 0.39, *SD *= 0.42). Finally, there was an interaction between reasoning and *acceptability of race‐based humor*:* F*(2, 504)* *= 51.57, *p *<* *.001, $\eta_{p}^{2}=.17$. Participants who thought the race‐based humor was okay were more likely to reference importance and less likely to reference welfare/rights and prejudice than were those who thought the race‐based humor was not okay (see Table 1), all *p*s < .001. Thus, participants who thought the humor was okay said things such as “it's not a big deal,” whereas those who thought the humor was not okay justified their response by citing welfare/rights (“it can hurt others’ feelings to tell jokes like that”) and prejudice (“because the joke he is saying is racist”).

**Table 1:** Justifications for Acceptability of Race‐Based Humor

	Not okay *M* (*SD*)	Okay *M* (*SD*)	Total
Welfare/rights	.51 (.42)^a^	.21 (.32)^a^	.41 (.42)^d^
Prejudice	.21 (.37)^b^	.01 (.07)^b^	.15 (.31)^d,e^
Importance	.13 (.27)^c^	.61 (.43)^c^	.28 (.39)^e^

Note.

Means with the same superscript differ significantly at *p *< .001.

### Expectations of Peer Responses to Race‐Based Humor

In order to test the hypotheses that, with age, participants would be less likely to expect peers to intervene in instances of race‐based humor and that there would be differences based on judgments of the *acceptability of race‐based humor*, a 4 (response: implicitly support, explicitly support, implicitly challenge, explicitly challenge) × 2 (age group: 8th, 10th) × 2 (acceptability of race‐based humor: okay, not okay) multivariate analysis of variance was conducted. Across the measures there were significant main effects of age group, *F*(4, 236)* *= 7.43, *p *<* *.001, $\eta_{p}^{2}=.11$, and acceptability of race‐based humor, *F*(4, 236)* *= 4.50, *p *= .002, $\eta_{p}^{2}=.07$, but no significant interactions (see Table 2). Follow‐up univariate ANOVAs were run for each potential response to further probe these main effects. There were age‐related effects for the following possible responses: explicitly challenge, *F*(1, 239)* *= 16.00 *p *<* *.001, $\eta_{p}^{2}=.06$, and implicitly challenge, *F*(1, 239)* *= 6.09, *p *= .01, $\eta_{p}^{2}=.025$ (see Table 2). In both cases, and consistent with hypotheses, 8th graders were more likely to expect their peers would intervene in these ways than were 10th graders. There were differences between those who thought the race‐based humor was okay and not okay for the following possible responses: explicitly support, *F*(1, 239)* *= 11.95, *p *= .001, $\eta_{p}^{2}=.04$; explicitly challenge, *F*(1, 239)* *= 6.37, *p *= .01, $\eta_{p}^{2}=.02$; and implicitly challenge, *F*(1, 239)* *= 10.66, *p *= .001, $\eta_{p}^{2}=.04$. Those who condoned the race‐based humor were more likely to explicitly support the joker and less likely to implicitly or explicitly challenge the joker (see Table 2).

**Table 2:** Expectations of Peer Responses to Race‐Based Humor

	Acceptability of race‐based humor		Age group		Total
	Not okay *M* (*SD*)	Okay *M* (*SD*)	8th *M* (*SD*)	10th *M* (*SD*)	*M* (*SD*)
Implicitly support	3.92 (1.30)	4.07 (1.18)	4.08 (1.23)	3.82 (1.30)	3.97 (1.27)
Explicitly support	2.69 (1.43)^a^	3.36 (1.38)^a^	2.87 (1.40)	2.93 (1.50)	2.90 (1.44)
Implicitly challenge	3.03 (1.16)^b^	2.43 (0.95)^b^	3.07 (1.09)^d^	2.56 (1.14)^d^	2.84 (1.13)
Explicitly challenge	3.70 (1.14)^c^	3.15 (1.14)^c^	3.83 (0.96)^e^	3.14 (1.29)^e^	3.52 (1.17)

Note.

1* *= *definitely would not* to 6* *= *definitely would*. Means with the same superscript differ significantly: ^a,b,e^ *p *< .001. ^c,d^ *p *< .01.

### Exclusion

It was hypothesized that participants who thought the humor was not acceptable would be more likely to expect an intervener to be excluded by peers but would be less likely to condone that exclusion than would participants who thought the humor was acceptable. To test this, a 2 (age group: 8th, 10th) × 2 (acceptability of race‐based humor: okay, not okay) × 2 (exclusion: likelihood, acceptability) ANOVA was conducted with repeated measures on the last factor. As expected, there was an overall effect for exclusion measure: *F*(1, 212)* *= 30.30, *p *<* *.001, $\eta_{p}^{2}=.12$. Participants were more likely to expect exclusion to occur (*M *= 2.94, *SD *= 1.64) than they were to judge exclusion as morally acceptable (*M *= 1.85, *SD *= 1.11). Furthermore, there was a significant interaction between exclusion and acceptability of race‐based humor, *F*(1, 212)* *= 26.39, *p *<* *.001, $\eta_{p}^{2}=.11$. As expected, participants who thought the race‐based humor was not okay were more likely to expect that exclusion would occur (*M *= 3.16, *SD *= 1.68, *p *<* *.01) and less likely to believe that exclusion was acceptable (*M *= 1.65, *SD *= 0.96, *p *<* *.001) than were participants who thought the race‐based humor was okay (likelihood: *M *= 2.40, *SD *= 1.40, acceptability: *M *= 2.32, *SD *= 1.31). Interestingly, no age‐related differences were found.

### Reasoning for Exclusion Measures

In order to test the hypothesis that participants would focus upon group functioning when justifying likelihood of exclusion and on welfare/rights when justifying acceptability of exclusion, a 4 (reasoning: welfare/rights likelihood of exclusion, welfare/rights acceptability of exclusion, group functioning likelihood of exclusion, group functioning acceptability of exclusion) × 2 (age group: 8th, 10th grade) ANOVA was conducted with repeated measures on the reasoning factor. Results supported the hypothesis: There was a main effect for reasoning, *F*(3, 522)* *= 60.98, *p *<* *.001, $\eta_{p}^{2}=.26$. Participants were much more likely to reference welfare/rights (*M *= 0.41, *SD *= 0.45) when justifying acceptability of exclusion than when justifying likelihood of exclusion (*M *= 0.10, *SD *= 0.28), *p *<* *.001. Participants were less likely to reference group functioning (*M *= 0.36, *SD *= 0.42) when justifying acceptability of exclusion than when justifying likelihood of exclusion (*M *= 0.70, *SD *= 0.44), *p *<* *.001. There were no effects for age group for either type of reasoning.

### Relations Between Expectations of Peer Responses to Race‐Based Humor and Exclusion

In order to test the hypothesis that likelihood of peer responses would predict likelihood of exclusion, multiple regression analyses were conducted on likelihood of exclusion. Likelihood of exclusion was used as the outcome variable, as we expected that participants’ expectations regarding peer responses would drive their expectations about exclusion. Variables for age in months, implicitly support, explicitly support, implicitly challenge, and explicitly challenge were entered into the regression model. In the first step, age in months was entered. In the second step, the implicitly support and explicitly support were entered. In the third step, implicitly challenge and explicitly challenge were entered. The final model was significant and accounted for 24% of the variance, *R* ^2^ **= .24, *F*(5, 201)* *= 2.49, *p *= .03. Implicitly challenge and explicitly challenge were the significant predictors. As participants’ expectations that their peers would implicitly challenge the race‐based humor increased, the likelihood of exclusion also increased (β = .25, *p *= .01). As participants’ expectations that their peers would explicitly challenge the race‐based humor increased, the likelihood of exclusion decreased (β = −.37, *p *= .01).

## Discussion

The aim of this study was to examine age‐related differences in how adolescents would evaluate a subtle form of discrimination (race‐based humor) to understand what types of responses to this discrimination they expected from their peers and to identify if they believed social exclusion would be a consequence for intervening in instances of race‐based humor. Findings suggest that generally adolescents did not support race‐based humor but that they also did not expect high rates of implicit and explicit intervention on the part of their peers, potentially because of concerns about social exclusion. Consistent with hypotheses, results revealed developmental differences: when told of an explicit group norm to accept humor, 10th‐grade participants judged the race‐based humor as more acceptable than did 8th‐grade participants. This finding may reflect an increasing sensitivity to group norms with age (Killen, Rutland, et al., 2013). Importantly, however, neither age group strongly endorsed race‐based humor: even the 10th‐grade participants’ evaluations of the race‐based humor hovered just below the midpoint of the scale. This reveals that, although younger adolescents are more likely to judge race‐based humor as morally unacceptable, adolescents at all ages recognize that such humor is problematic. This was reflected in participants’ reasoning: younger adolescents focused more centrally on issues of welfare/rights, whereas older participants focused on importance, arguing that “a joke isn't a big deal.” This apathy on the part of older adolescents is particularly concerning, given the significant negative consequences even subtle forms of discrimination can have for victims (Huynh, 2012).

Participants who condoned the race‐based humor were also much more likely to suggest that the act was of low importance, whereas those who judged the humor to be unacceptable made more reference to welfare/rights as well as to prejudice. The finding that participants who supported the race‐based humor judged it to be unimportant indicates that race‐based humor does function similarly to microaggressions (Huynh, 2012; Nadal, 2011) and is perceived by many to “not matter much,” as one participant said. Most adolescents did not overtly support race‐based humor, and they considered such humor through a unique lens, weighing both moral issues (welfare/rights, and prejudice) and issues surrounding importance. Importantly, however, even though more than half of the participants (*N *= 176) judged the race‐based humor as not acceptable, the only response that crossed the midpoint of the scale was to implicitly support the joker by staying with the group and not acting. This finding is concerning, given research that shows how powerful bystander intervention is in halting bullying (Hawkins et al., 2001) and how lack of bystander intervention can cement norms for discriminatory behavior (Aboud & Joong, 2008). However, it is not surprising that participants were likely to expect their peers to implicitly support the race‐based humor, as inaction is a far too common response to this type of transgression. This finding highlights the importance of developing school‐based interventions that reiterate the serious nature of and negative outcomes associated with seemingly trivial or everyday instances of discrimination occur among peers. Additionally, it is important that such interventions highlight the positive impact that intervention can have. Movement toward creating interventions that outline the seriousness of such subtle forms of race‐based bullying for adolescents is especially important given that ignoring such discrimination can normalize behavior such as this (Aboud & Joong, 2008) and could lead to more explicit discrimination resulting in more widespread problems.

The age‐related differences found in evaluations of the acceptability of the humor were also reflected in participants’ expectations regarding peer responses. High school adolescents were less likely to expect peers to intervene in instances of discriminatory race‐based humor than were middle school adolescents. This may be explained by the increasing influence of the peer group across adolescence (Brechwald & Prinstein, 2011) and the greater focus on the importance of group functioning with age (Horn, 2003). This is an important finding, as bullying prevention programs typically focus on the elementary and middle school period (see Evans, Fraser, & Cotter, 2014, for a review of recent interventions). However, the present evidence suggests that future research aimed at encouraging adolescents to challenge discriminatory peer group norms or behavior and to intervene in instances of bullying should also target high school students. Research with children up to age 11 documented age‐related increases in thinking of possible intervention strategies to respond to bullying (Rock & Baird, 2012). The current findings suggest that although children may be able to generate a wider variety of possible interventions or bystander responses with age, as they enter adolescence they may curb their expectations about which strategies will actually be used.

Participants who thought the race‐based humor was acceptable were also less likely to expect their peers to intervene than were those who thought the race‐based humor was unacceptable, supporting the suggestion by Aboud and Joong (2008) that expectations regarding intervention and a norm for acceptability are correlated. Specifically, participants who judged race‐based humor as unacceptable were more likely to expect a dissenting member to implicitly and explicitly challenge the discrimination, whereas participants who judged race‐based humor as acceptable were more likely to expect a dissenting member would explicitly support the joke by laughing. Those who do not support race‐based humor, therefore, expect their peers to take an active role in challenging such discriminatory behavior and expect their peers to seek support in a range of different ways. The finding that participants assumed similarity between the in‐group and the self is consistent with previous research on children's in‐group bias and social projection (Abrams, 2011).

These results also complement and extend prior research on bystander intervention (Abbott & Cameron, 2014; Palmer et al., 2015; Salmivalli et al., 2011) by revealing that participants’ moral judgments regarding the discriminatory acts are related to their expectations of bystander interventions. Moreover, previous research on bystander interventions has not yet focused on intervening when a member of your own group is engaging in the harmful behavior and when your group norm supports such behavior. Consistent with adult research showing that awareness of explicit racism by in‐group members may raise a moral alarm (Abrams et al., 2014), the research reveals that adolescents who recognize the problematic nature of race‐based humor are more likely to expect their peers to intervene even in contexts where issues surrounding loyalty to the group and adherence to group norms are paramount. Just as was found in research assessing deviating from group norms surrounding resource allocation (Killen, Rutland, et al., 2013), the current research reveals that some adolescents do not support their group norms and extends this work by revealing that, in circumstances where the group norm perpetuates discriminatory behavior, they do expect peers to intervene. These findings suggest an interplay between peer‐level norms and generic norms (those which are held by a society or institution, such as a school, more generally), in concert with the proposed interplay outlined by Nesdale and Lawson (2011). Future research should continue to examine developmental patterns in how group norms interact with generic social or school norms, which may discourage discriminatory behavior. Specifically, the current findings suggest that adolescents may, at times, prioritize peer group norms, even when those perpetuate discrimination, which is counter to generic social or school norms.

One possible account of the preceding findings is that adolescents who are likely to intervene are somehow less aware of or less sensitive to group norms. However, the findings suggest instead that these adolescents take a principled stance that fully recognizes the contrast between intervening on the basis of a generic social norm, which discourages racism and upholding immediate group norms (implied by shared humor). Specifically, participants’ moral judgments about engaging in discriminatory race‐based humor drove participants’ expectations about the consequences for intervening. Participants who thought it was not okay to use race‐based humor were *more* likely to expect exclusion for challenging such humor. They also thought that this exclusion would be less acceptable than those who condoned race‐based humor. Prior research from social domain theory has documented that judgments about the acceptability of exclusion are related to judgments of favorability toward deviant members (Hitti et al., 2014). These results extend this prior research from social domain theory revealing that judgments about the acceptability of an act that aligns with group norms (the race‐based humor) are related to evaluations of exclusion of someone who challenges that act. This is a novel finding that reveals the depth of the complexity of adolescents’ social reasoning.

Moreover, this is the first research to reveal that moral judgments about the act that a group member challenges (race‐based humor) drive evaluations of both the likelihood of exclusion and the acceptability of exclusion. Measuring both likelihood and acceptability of exclusion was a novel feature of the current study. Consistent with subjective group dynamics theory (Marques et al., 2001), the findings reveal that participants who reject race‐based humor (i.e., who are willing to deviate from a salient group norm) may be highly sensitized to that norm and are more likely to think that peers who intervene will be excluded for challenging the group and that this exclusion is less acceptable than participants who condone race‐based humor. Moreover, the reasoning results support and extend social domain theory by demonstrating how distinct these judgments of acceptability and likelihood are: Adolescents reference welfare and rights much more frequently when evaluating the acceptability of exclusion, and group functioning much more frequently when evaluating the likelihood of exclusion. Thus, adolescents recognize the complexity of decisions regarding exclusion, and perceive that while an act such as using racist humor may be wrong for moral reasons, it may be sustained or tolerated as a result of group dynamics.

Therefore, rather than being oblivious to the norm, these adolescents are actively rejecting it, with full awareness of the implications for peer exclusion. Although much prior research has documented that children and adolescents judge exclusion to be unacceptable (Killen & Rutland, 2011), their group nous makes them well aware that peers may be motivated to exclude those who deviate from their group's norms (Abrams et al., 2009). What is concerning, however, is that participants who were more likely to expect their peers to implicitly challenge the discrimination were more likely to expect that social exclusion may result from such a challenge, whereas those who were more likely to expect their peers to explicitly challenge the discrimination were less likely to expect that social exclusion may occur. This suggests that adolescents who recognize that social exclusion is a strong possibility for those who intervene also understand that their peers may take less overt paths to resistance, and engage in implicit, rather than explicit challenges. Although implicit challenges such as talking to an adult or a friend afterward are also important forms of intervention, they may not send as clear and immediate a message to the potential victims of the discrimination, to the joke teller and to other bystanders that such discrimination is unacceptable and will not be tolerated.

Overall, this study contributes to our understanding of adolescents’ evaluations of discriminatory group norms, those that perpetuate race‐based humor, and their expectations regarding peer responses to race‐based humor and the consequences for intervening in such discriminatory behavior. The current study does have limitations, however. First, the participants were primarily European American from largely ethnically homogenous schools. Research indicates that intergroup contact and heterogeneous school composition is related to more positive intergroup relations (McGlothlin & Killen, 2010). Thus, future research should sample participants in both homogenous and diverse school settings, as the school diversity context may impact adolescents’ evaluations. Research on cross‐group friendships and intergroup contact suggests the positive benefits of such friendships for ethnic minority and majority students (Bagci et al., 2014; Graham et al., 2014; Tropp, O'Brien, & Migacheva, 2014; Tropp & Prenovost, 2008). Future research should examine if adolescents with more cross‐group friendships are more likely to intervene in instances of discrimination. Also, as the schools sampled for this study were not perfectly matched in terms of socioeconomic status, future research should investigate whether differences by socioeconomic status exist in evaluations of race‐based humor. Furthermore, the current study assessed participants’ expectations about their peers’ reactions to race‐based humor but did not measure participants’ own intervention behaviors. Extending this research by examining peer expectations and individual behaviors (i.e., if adolescents do challenge their own peer group when their group holds discriminatory group norms) would provide insights into these complex peer processes. Finally, we did not find any differences between evaluations of African American and Latino targets. Extensions of this research should include targets from different out‐groups, such as Arab Americans, or in different cultural contexts. This is an important new direction as research demonstrates that not all intergroup contexts are evaluated in the same way (Mulvey, Hitti, Rutland, Abrams, & Killen, 2014) and discrimination occurs in different ways and toward different out‐groups across our changing global landscape. Additionally, although the current study focused on a subtle form of discrimination, race‐based humor, we recognize that sensitivity to racism may not be the same as sensitivity to other domains of prejudice (cf. Abrams, Houston, Van de Vyver, & Vasiljevic, 2015). Therefore, future research should investigate other targets of discrimination as well as expressions that are either more or less overt forms of discrimination than humor. For example, it may be that adolescents are more likely to challenge more overt forms of discrimination as the negative consequences of more direct forms of discrimination are more obvious. The results from the current study reveal variation in adolescents’ evaluations of race‐based humor and demonstrate that their judgments of race‐based humor influence their expectations about intervention and the consequences for intervention.

The results have significant implications for understanding peer group dynamics, discrimination, moral judgments, and social cognition. The findings reveal that adolescents struggle to balance the wrongfulness of discrimination and the social pressure of groups. Participants who judge race‐based humor as unacceptable are more likely to expect their peers to intervene, but they are also more likely to expect that those who challenge the discrimination will face social exclusion. Concerns over social exclusion may serve as a significant obstacle to adolescents who would like to resist race‐based discrimination and intervene by challenging discriminatory group behaviors. The findings suggest that interventions targeting racial/ethnic discrimination should include a focus on more subtle forms of discrimination, such as race‐based humor and that such interventions could also provide guidance or training to students on different ways that they might intervene, including both implicit and explicit interventions, and on the value of building cross‐group friendships with those who do not share one's own ethnic or racial group. The results indicate that teachers, parents, group leaders, and peer groups should seek ways to encourage adolescents, especially high school students who are often overlooked in bullying interventions (Evans et al., 2014), to think critically about group norms and behaviors and to actively challenge discriminatory, hurtful behavior.
